# The Role of Mesenchymal Stromal Cells in the Treatment of Bronchopulmonary Dysplasia: A Multi‐Prong Approach for a Heterogeneous Disease

**DOI:** 10.1002/cph4.70038

**Published:** 2025-08-25

**Authors:** Marc‐Olivier Deguise, Bernard Thébaud

**Affiliations:** ^1^ Division of Neonatology, Department of Pediatrics Children's Hospital of Eastern Ontario Ottawa Ontario Canada; ^2^ Children's Hospital of Eastern Ontario Research Institute Ottawa Ontario Canada; ^3^ Department of Obstetrics, Gynecology and Newborn Care The Ottawa Hospital—General Campus Ottawa Ontario Canada; ^4^ Sinclair Centre for Regenerative Medicine The Ottawa Hospital Research Institute Ottawa Ontario Canada; ^5^ Faculty of Medicine, University of Ottawa Ottawa Ontario Canada

## Abstract

Acute lung injury can be a devastating ailment leading to death in patients of all ages. In preterm neonates, lung injury is unique and unlike what is seen in pediatric and adult populations. The physiology behind the acute lung injury endured in developing lungs and the chronicity of harmful stimuli vastly distinguish how bronchopulmonary dysplasia (BPD), the most common complication of prematurity, settles in as a chronic lung disease with lifetime sequelae. Despite being recognized for over 50 years, BPD continues to puzzle the world of neonatology with a shifting phenotype that parallels improvement in neonatal care. The improved understanding of BPD's far‐reaching and long‐term consequences on the lung and other organs highlights the need to find effective interventions, making it a priority of neonatal research. In this review, we provide an overview of BPD and its associated consequences. Then, we examine the biological premises for mesenchymal stromal cells as a promising therapy, reviewing current translational efforts, challenges, and future directions toward bringing mesenchymal stromal cell therapy to BPD patients.

## 1 Bronchopulmonary Dysplasia: A Primer

### 1.1 An Evolving and Heterogeneous Disease of Lung Development

Bronchopulmonary dysplasia (BPD) is a devastating and relentless complication of prematurity with rates that have remained steady over the last 30 years (Stoll et al. 2015; Horbar et al. 2024). This parallels the increased survival seen in the most premature infants (Horbar et al. 2024; Domellof and Jonsson 2018; Edwards et al. 2024; Kono et al. 2021). It is the most common complication of prematurity, affecting at least one‐third to half of extremely low gestational age neonates (ELGANs—born less than 28 weeks of gestation) (Stoll et al. 2015; Thébaud et al. 2019; Lawn and Kinney 2014). The population of preterm infants and the harmful stimuli leading to BPD pathogenesis have changed considerably with the evolution of neonatal care (Stoll et al. 2015); so has the BPD phenotype (reviewed in Jobe 1999; Philip 2012; Baraldi and Filippone 2007). The initial description of BPD in the 1960s highlighted a severe destructive phenotype of the pre‐established lung architecture marked by important remodeling and fibrotic changes with a heterogenous multi‐cystic appearance of the lung, mostly seen in very/late preterm infants (Philip 2012). This injurious process was driven by high oxygen exposure and harsh invasive mechanical ventilation (Philip 2012). This phenotype is now termed “old” or “classical” BPD (Baraldi and Filippone 2007; Cassady et al. 2020). With the advent of new neonatal respiratory care strategies (antenatal steroids, exogenous surfactant, caffeine, “gentle” ventilation), BPD has transitioned to a developmental disruption of lung growth. Termed the “new” BPD in the 1990s (Jobe 1999; Philip 2012; Baraldi and Filippone 2007), it is commonly seen in the youngest (22–25 weeks gestational age [GA]) ELGANs, with a pathology marked by reduced complexity of the lung architecture, with fewer and variable alveolar components associated with remodeling of the vasculature. These ELGANs have more immature and fragile lungs with a consequent risk of BPD reaching up to 80% (Younge et al. 2017; Norman et al. 2023), for which there are limited therapeutic interventions (Thébaud et al. 2019).

### 1.2 Same‐Same but Different: Is BPD Best Defined by Respiratory Management as a Persistent and Sole Diagnostic Criterion?

Unlike its first description based on the pathological findings of the lungs (Northway Jr. et al. 1967), BPD diagnosis has since continued to rely on the level of respiratory support at a specific time point rather than the underlying pathogenic processes driving the disease. This diagnostic method occurring late in the ELGANs' postnatal life (sometimes 8–14 weeks after birth) comes at the expense of missed opportunities to act at the earliest moment in the pathogenesis of the “evolving BPD”. For example, histological changes in line with BPD and pulmonary hypertension are often present before the clinical BPD diagnosis (Stone et al. 2022). Some ELGANs may die of severe pulmonary disease or respiratory failure before the diagnosis of BPD can even be made, raising the questions whether they should be considered early lethal BPD (Steinhorn et al. 2017). Clinicians must rely on early predictive factors determining BPD diagnosis for clinical trial enrollment, which are imperfect. Furthermore, a diagnosis based on respiratory support may misclassify premature infants who have added contributions from other pathologies (such as vocal cord paralysis, upper airway obstruction/narrowing, or neurological impairments, among others) rather than the sole contribution of the altered lung development. Thus, BPD diagnosis has been a particular topic of contemplation with numerous suggested definitions provided over the past decades. Jensen et *al*. recently systematically studied 18 definitions to best predict death or long‐term respiratory/neurodevelopmental outcomes associated with BPD with the hope to accurately risk‐stratify this population of patients (Jensen et al. 2019). The resulting definition relies on the intensity of respiratory support at 36 weeks regardless of the oxygen fraction required (Jensen et al. 2019). This definition suggests BPD as a rather homogenous disease process, with patients having a similar disease phenotype. Despite a continuum of severity established by this definition, it would be suggestive that therapies targeting BPD may or should perform as a “one‐size fits all”. However, BPD is a very heterogenous entity, affected by factors that differ from patient to patient. As a striking example, BPD patients with and without pulmonary hypertension have different disease progression, the former associated with poorer outcomes (Altit et al. 2019; Aoyama et al. 2021). Recent studies suggest that numerous BPD phenotypes are potentially recognizable (Pierro, Van Mechelen, et al. 2022), though this concept is not commonly applied in clinical practice. Our understanding of BPD patients and their diagnosis will eventually have to rely on additional aspects beyond their respiratory support at 36 weeks corrected gestational age (cGA) (Day and Ryan 2017). Delineating their course, either via biomarkers (blood, urine, tracheal secretions), imaging (such as ultrasound and magnetic resonance imaging), clinical trajectories (such as those previously defined; Laughon et al. 2009; Laughon, Bose, et al. 2011), response to known therapeutics (dexamethasone) (Steinhorn et al. 2017) and ''omics'' approaches (including single cell transcriptomics; Hurskainen et al. 2021; Travaglini et al. 2020) aided by artificial intelligence (Husain et al. 2024) will offer a path toward identification of BPD endotypes/phenotypes (Pierro, Van Mechelen, et al. 2022; Wu et al. 2020) to tailor personalized management strategies.

### 1.3 50 Years of BPD Management: Where Are We Now?

**FIGURE 1:**
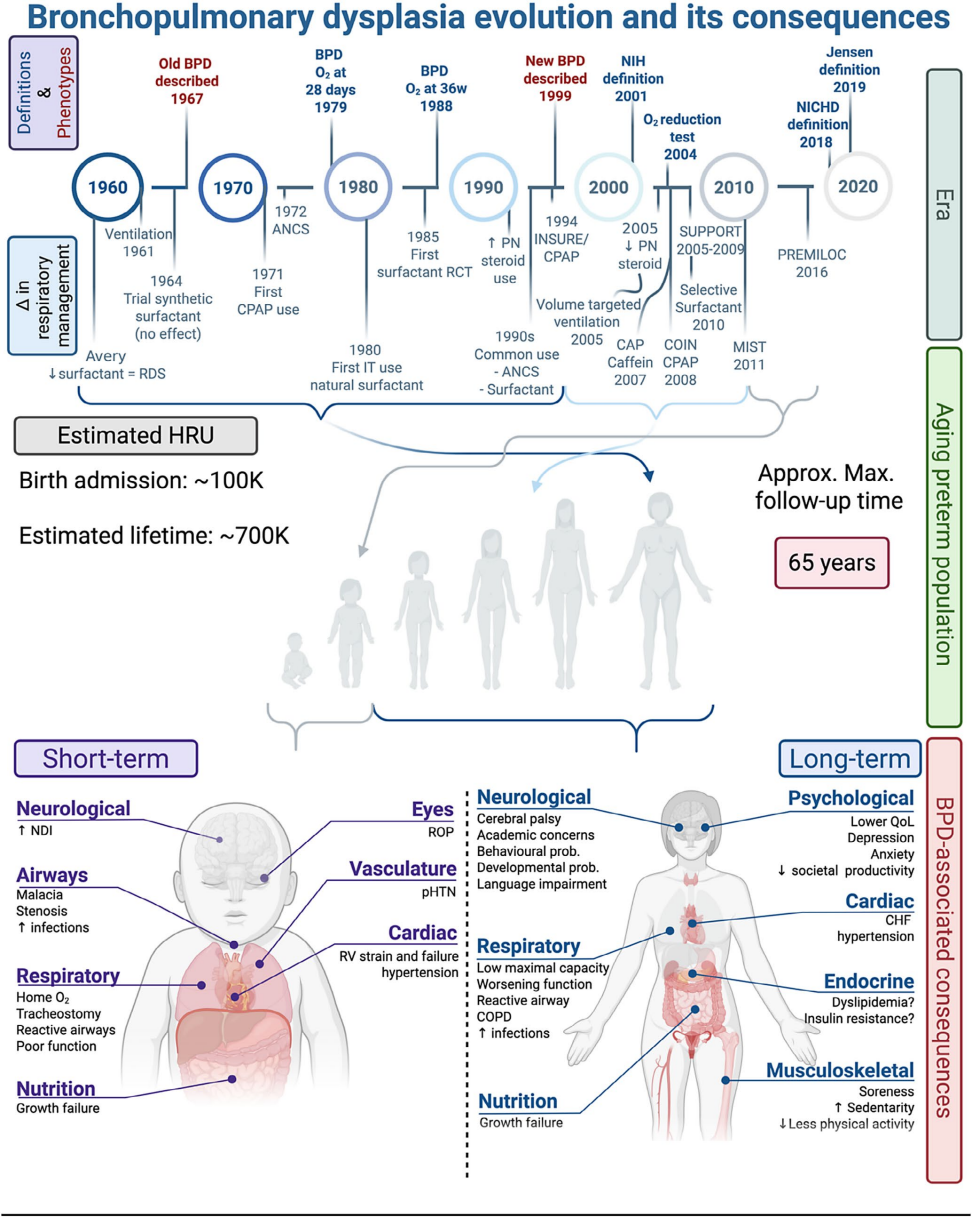
Timeline of the evolution of BPD and respiratory care, highlighting the currently aging preterm population from each era, displaying both acute and long‐term consequences of BPD. Schematic timeline presenting the evolution of BPD through its diagnostic definitions and phenotypes in relation to the change in respiratory care over the years. Note that the schematic only presents prominent definitions and changes in management. Definitions have been reviewed extensively elsewhere (Thébaud et al. 2019; Wu et al. 2024; Ibrahim and Bhandari 2018). It is not meant to be an exhaustive representation of BPD definitions reported nor of the various changes in management in neonatology. Aging BPD is shown in relation to the changes in respiratory care during their era, highlighting that most reports of long‐term consequences of BPD currently published are a representation of the “old BPD” phenotype, with yet an undefined long‐term progression of the “new” BPD phenotype during most of adulthood. The estimated longest follow‐up reported for patients with BPD was last described as 53 years old (Bui et al. 2022) but could theoretically be as old as 65 years of age. Estimated healthcare resources utilization is also featured (Lapcharoensap et al. 2018; Van Katwyk et al. 2020). Short‐term (left) and long‐term (right) co‐morbidities associated with BPD. ↓, reduce; ↑, increase; Δ, change; ANCS, antenatal corticosteroid; Approx., approximative; BPD, bronchopulmonary dysplasia; CHF, congestive heart failure; COPD, chronic obstructive pulmonary disease; CPAP, continuous positive airway pressure; HRU, health resource utilization; IMV, invasive mechanical ventilation; INSURE, INtubate‐SURfactant‐Extubate; IT, intratracheal; IUGR, intra‐uterine growth restriction; Max, maximum; MIST, minimally invasive surfactant administration; NDI, neurodevelopmental impairment; NEC, necrotizing enterocolitis; NICHD, National Institute of Child Health Disease; NIH, National Institute of Health; O_2_, oxygen; pHTN, pulmonary hypertension; PN, post‐natal; Prob, problem; QoL, quality of life; RCT, randomized clinical trial; RDS, respiratory distress syndrome; ROP, retinopathy of prematurity; RV, right ventricle; w, week; Y, year. PREMILOC, COIN, SUPPORT, CAP refer to short names of well‐known trials in the field of neonatology. Created in BioRender. Deguise, M. (2025) https://BioRender.com/onk2zj1 (Adaptations of numerous BioRender (2025) templates: Human Ages Timeline, Lugano, G ‐ Congenital Diaphragmatic Hernia, Delrose, N. Cancer‐Associated Comorbidities).

Despite managing premature infants with evolving BPD for many decades, neonatologists still have minimal therapeutic options for this patient population (DeMauro 2021). Current management relies on minimizing what we know to be harmful to the developing lung (i.e., mechanical ventilation, oxygen, etc.). Numerous innovations in the respiratory care of the ELGAN have emerged. The introduction of surfactant (Dunn et al. 1991), widespread use of non‐invasive ventilation (Morley et al. 2008; Dunn et al. 2011; SUPPORT Study Group of the Eunice Kennedy Shriver NICHD Neonatal Research Network 2010b), routine use caffeine (Schmidt et al. 2006), antenatal steroids (JAMA 1995; Carlo et al. 2011; Mori et al. 2011), variations of supraphysiologic postnatal steroid administration protocols (Doyle et al. 2006, 2025), and the prophylactic administration of physiologic hydrocortisone doses for relative corticosteroid insufficiency (Baud et al. 2016; Baud and Lehert 2024) have improved, to various extents, the outcomes of ELGANs (DeMauro 2021) (Figure 1). Some of these strategies have shown reductions in BPD rates when studied in isolation (reviewed in Thébaud et al. 2019; DeMauro 2021), but the agglomeration of these strategies within real‐world neonatal applications and population studies has led to minimal changes in BPD rates (Stoll et al. 2015; Horbar et al. 2017, 2024). It may not be feasible to completely limit lung injury in the sicker and increasingly more premature infants (22–24 weeks GA) due to developmental limitations. Without a better understanding of BPD pathogenic processes, we may not curb the effect of BPD on our most susceptible ELGANs.

### 1.4 Short‐ and Long‐Term Consequences of BPD: More Than Just the Lungs

#### 1.4.1 The Early Sequelae Affecting BPD Infants' Development

BPD is a particularly important morbidity for ELGANs. Both short‐ and long‐term associated consequences arise from multiple other organ systems beyond the respiratory system. Other reviews have covered this topic in significantly more details (DeMauro 2021; Homan and Nayak 2021; Yaremenko et al. 2024). During the neonatal intensive care unit (NICU) stay, the evolution of patients who will develop BPD involves longer exposure to invasive ventilatory support and enhanced intensity of care (Jensen, Edwards, et al. 2021). This puts BPD patients at higher risk of contracting infections such as ventilator‐associated pneumonia and sepsis (Huang et al. 2024). Those who develop late‐onset sepsis are more at risk of having home oxygen or a tracheostomy (Flannery et al. 2022). BPD also increases the risk of pulmonary hypertension and right heart strain (Thébaud et al. 2019; Durlak and Thébaud 2024). Poor oxygenation capacity in BPD can lead to a chronic, variably intermittent state of hypoxia, which may be underappreciated and have unmeasured consequences. Among them, intermittent hypoxia (IH), primarily driven by immature respiratory control affecting all preterm infants, disproportionately impacts BPD patients (Jensen, Whyte, et al. 2021; Raffay et al. 2019) due to their poor respiratory function and reserve (briefly reviewed in Martin et al. 2015). Other BPD pathogenesis/characteristics, such as inflammation, airway and arterial reactivity, and increased airway resistance, may worsen induction and recovery from IH episodes (Martin et al. 2015). IH adversely impact neurodevelopment (Jensen, Whyte, et al. 2021; Abu Jawdeh 2017), airway hyperreactivity (Raffay and Martin 2020), pulmonary hypertension (Gentle et al. 2023) and is associated with the development of cardiovascular risk factors such as hypertension (Martinez et al. 2025). Poor oxygenation is also associated with higher levels of oxygen administration, which can be directly associated with the development of retinopathy of prematurity and possibly blindness in severe cases (Hellström et al. 2013). Evolving lung injury and ongoing reliance on invasive mechanical ventilation often lead to the use of postnatal steroids to facilitate extubation, which is itself associated with poor neurodevelopmental outcomes based on historical studies (DeMauro 2021; Doyle et al. 2005, 2025; Hay et al. 2023). Moreover, BPD is intertwined with the potential for growth failure (reviewed in DeMauro 2021; Homan and Nayak 2021). The severity of BPD may require patients to go home on oxygen or with a tracheostomy, which further complicates their journey (Annesi et al. 2021). BPD is associated with more procedures, longer use of respiratory support, ultimately leading to significantly longer initial hospital stay (Lapcharoensap et al. 2018). After graduating from the NICU, BPD patients appear to be more susceptible to infections, with increased risk of hospital readmission, respiratory support, and need for intensive care (Lapcharoensap et al. 2018, 2020).

#### 1.4.2 Long‐Awaited: Demystifying Long‐Term Outcomes With the Evolving, but Distinct, BPD Populations

Considering our knowledge on the long‐term outcomes of BPD patients, possible caveats arise. The constantly evolving BPD definition/phenotype and respiratory care of patients over the last 50 years generate potentially diverse and perhaps distinct populations, akin to the differentiation of BPD (old vs. new) between eras. While there is likely to be overlap between these BPD populations, it is unclear whether the knowledge generated in adults with BPD representative of the “old BPD” phenotype (now older adults) can be extrapolated to the “new BPD” phenotype (now young adults). Comparison of functional outcomes between BPD eras is limited. With the description of the “new BPD” phenotype in the 1990–2000s, published studies on the long‐term outcomes of patients with the “new BPD” phenotype would be reasonably restricted to adults in their thirties (Figure 1). In addition, the long‐term outcomes likely under‐represent severe BPD patients, especially if afflicted with severe comorbidities (such as severe cerebral palsy). In general, it is appreciated that clinical trials tend to enroll healthier patients with favorable socioeconomic status (Shaikh et al. 2024; Wright et al. 2006; Rich et al. 2012). In the field of neuromuscular disease, the study burden and complexity as well as travel time were the most important reasons for declining participation (Naarding et al. 2020). It is reasonable to consider that the functional status of severe BPD patients may be limiting their participation in such studies, thereby leading to selection bias due to an inflated representation of mild to moderate BPD disease. Similar research about potential selection bias of BPD patients in long‐term studies is not imminently evident/available but should be sought in the future. Certainly, this phenomenon is seen in short‐term clinical trials for the infants most at risk of BPD. A recent meta‐analysis focused on the representation of clinical trials of the last decade in neonatology identified that only one‐fourth of studies included infants less than 24 weeks, with an overall representation of 1.4% of all patients (Pavlek et al. 2021). To ensure proper interpretation of long‐term outcomes, careful evaluation of the cohort birth era and current age may help identify whether “old BPD” versus “new BPD” differ in their epidemiological characteristics, prognosis, and long‐term outcomes.

#### 1.4.3 Doubling Down: BPD Restricts Attainment of Full Respiratory Function Potential and Precipitates Its Decline

The theory of lung function trajectory, stipulating that a peak lung function is achieved by early adulthood followed by a progressive deterioration (Lange et al. 2015; Krishnan and Martinez 2018; Stocks et al. 2013) is well accepted. BPD and prematurity may limit the maximal respiratory potential and accelerate its decline (reviewed in Mcgrath‐Morrow and Collaco 2019). BPD adults show reduced forced expiratory volume in 1 s (FEV_1_), forced vital capacity (FVC), ratio of FEV_1_/FVC, forced expiratory flow at 25%–75% of FVC (FEF_25%‐75%_), diffusing capacity of the lung for carbon monoxide (DLCO), often worse than asthmatic peers (Um‐Bergstrom et al. 2019) and seemingly associated with the BPD severity (Gough et al. 2014). Clinically, aging BPD patients are often thought to have reactive airway disease akin of asthmatics given increased wheezing, breathlessness, and coughing early in childhood (Baraldi and Filippone 2007). Despite being mislabeled as “physician diagnosis of asthma” (Gough et al. 2014), they display preferential small airway involvement (Um‐Bergstrom et al. 2019), with variable modest to minimal response to standard asthma therapies (Cassady et al. 2020). Of interest, a recent report highlights the role of BPD among various spirometry profiles in a large preterm cohort (Cousins et al. 2023). BPD patients are over‐represented in the obstructive spirometry profiles but not in other profiles such as prematurity‐associated preserved ratio of impaired spirometry (pPRISm) or prematurity‐associated lung dysanapsis (Cousins et al. 2023). Emerging evidence suggests a potential role for CD8 cytotoxic T‐cells (Um‐Bergstrom et al. 2022) in ongoing lung inflammation, with low eosinophils numbers (unlike asthmatic lungs) (Galderisi et al. 2019), which may contribute to the depreciating lung function over time in BPD. Interestingly, this can resemble pathogenic processes seen in chronic obstructive pulmonary disease (COPD), tying the concept that BPD may precipitate an early COPD phenotype (Baraldi and Filippone 2007; Cassady et al. 2020; Mcevoy and Aschner 2015). Moderate (32–34 weeks GA) to very preterm (28–32 weeks GA) born adults (“old BPD”) are at increased risk for COPD in their fifth decade when compared with term‐born adults (Bui et al. 2022). In addition, the poor baseline respiratory capacity foster grounds for more frequent and severe respiratory illnesses during childhood which can additively impact the respiratory lung trajectory (Stocks et al. 2013).

#### 1.4.4 Beyond the Lungs: Systemic Sequelae of BPD

BPD may present as separate clinical entities in adulthood, spanning an asthma‐like, obstructive, or pulmonary hypertensive phenotype (Cassady et al. 2020). A proportion of BPD patients will be identified with high pulmonary pressure during their NICU stay; two‐thirds of patients will have resolving pulmonary pressure during childhood, while one‐third of patients will have persistently high pulmonary pressure (Altit et al. 2019). This phenotype will present with increased resting vascular tone and exaggerated vasoconstriction during hypoxic stimuli, with consequent right‐to‐left shunting and eventual signs of reduced cardiac function in later stages (Cassady et al. 2020; Homan and Nayak 2021; Hansmann et al. 2021; Lewandowski et al. 2013). It is unsurprising to find that BPD patients have altered exercise capacity over the course of their life (reviewed in Homan and Nayak 2021; Yaremenko et al. 2024; Malleske et al. 2018). The cardiorespiratory system is likely a major contributor to this effect, in addition to the recent finding of reduced muscle strength in adults born premature, worse in BPD patients (Deprez et al. 2024). While BPD patients are often not segregated in many association studies between preterm and term‐born patients in relation to their risk for adult ailments, their worsened respiratory outcomes, exercise capacity, and variable hypertensive phenotype indirectly put them at the forefront of increased risks of disease among the aging preterm population. Preterm infants are at higher risk for hypertension, increased fat mass, and less convincingly insulin resistance and dyslipidemia (Lewandowski et al. 2013; Markopoulou et al. 2019). Arguably, one of the most common associations with BPD is the increased risk of adverse neurodevelopmental outcomes (DeMauro 2021). BPD has been consistently linked to cerebral palsy, cognitive, intellectual quotient, academic, visual‐motor integration impairments, with poor school performance attainment, among others (the full scope of this topic has been reviewed elsewhere; DeMauro 2021).

Altogether, BPD has far‐reaching consequences impeding their wellness and anticipated developmental potential later in life. It is not surprising that it can have an important impact on the quality of life of BPD patients (Homan and Nayak 2021; Gough et al. 2014). As such, early interventions to tackle BPD pathogenesis is imperative.

### 1.5 Deciphering the Progressive Pathogenesis of BPD

#### 1.5.1 Mechanistic Elucidation of BPD Complicated by the Various Stages of Lung Development

Understanding the underpinnings of BPD is challenging. Compared with a 28‐week ELGAN (in the saccular stage of lung development), the same insults in a 22‐week ELGAN (in the canalicular stage) will lead to alterations at different levels of organogenesis (e.g., secondary septation vs. primary septation, respectively), affecting the underlying pathogenic processes and the extent of the molecular injury. Heterogeneity also stems from each ELGAN's unique pre‐ and post‐natal course, making BPD's chronic injury process variable. Yet, clinically, they may be labeled with the same “BPD” diagnosis of similar severity at 36 weeks cGA using current diagnostic criteria.

#### 1.5.2 The “In Utero” Environment: Establishing Susceptibility to BPD From the Earliest Timepoint

**FIGURE 2:**
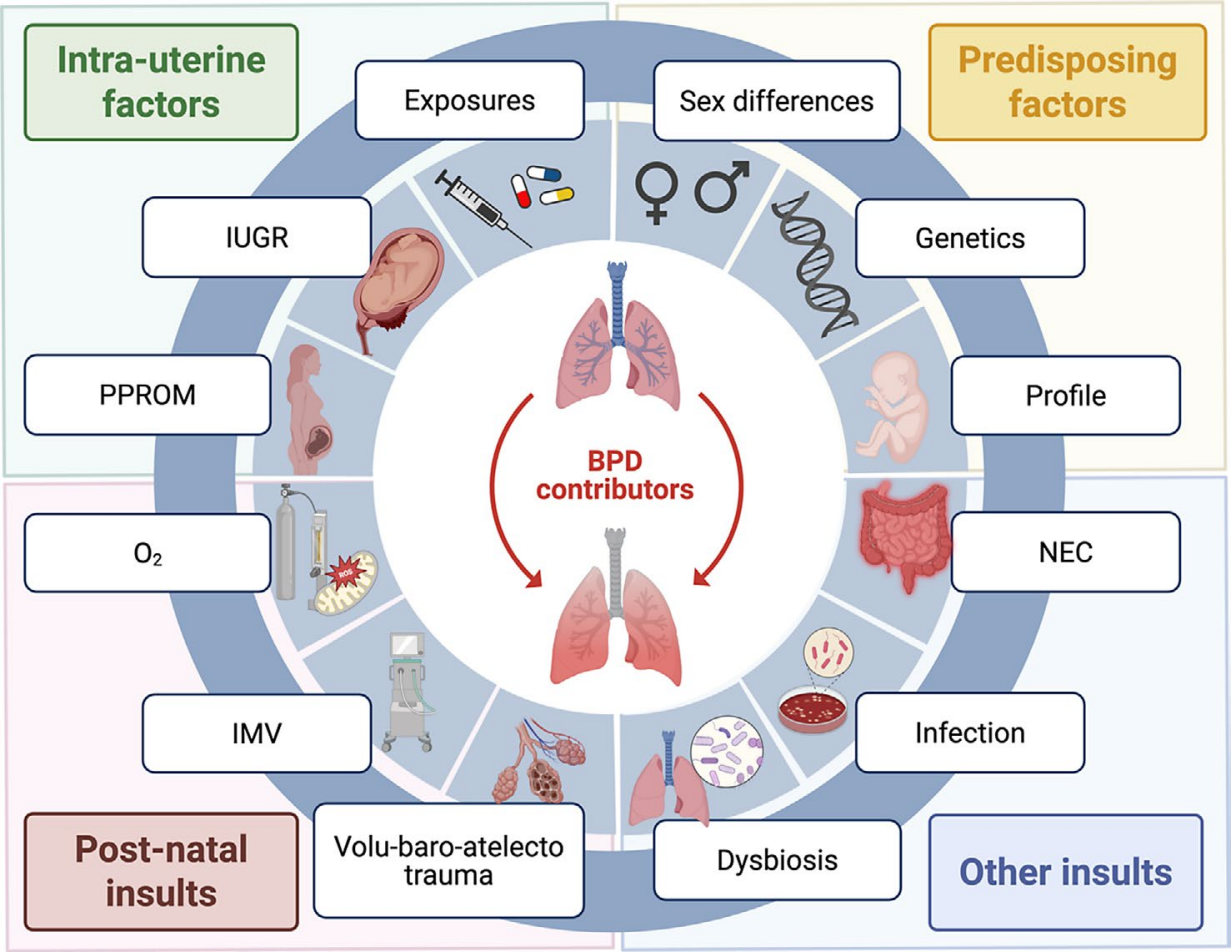
The various components contributing to the development and severity of BPD. Schematic representation of selected identified (not exhaustive) contributors or risk factors contributing to the development of BPD divided in four categories (Intra‐uterine factors, predisposing factors, post‐natal insults, other insults). BPD, bronchopulmonary dysplasia; IMV, invasive mechanical ventilation; IUGR, intra‐uterine growth restriction; O_2_, oxygen; NEC, necrotizing enterocolitis; PPROM, prolonged premature rupture of membrane. Created in Biorender. Deguise, M. (2025) https://BioRender.com/swwb7j7 (modified from Biorender (2025) Gut‐brain axis regulators template).

Even before their first breath, ELGANs' lungs are at the mercy of prenatal in utero exposure (Figure 2—*Pre‐disposing factors*). Detrimental exposure to placental insufficiency leading to poor growth (Pierro, Villamor‐Martinez, et al. 2022), toxic substances (i.e., tobacco; Steinhorn et al. 2017) (reviewed in Kajekar 2007), and maternal infectious processes (such as chorioamnionitis) are risk factors for BPD (Berger and Bhandari 2014). Likewise, oligohydramnios due to prolonged premature rupture of membrane, a common precursor of prematurity, can lead to lung hypoplasia, further interfering with normal lung development before birth (Plosa and Sucre 2024). Compelling evidence highlights inflammatory triggers as important contributors to BPD pathogenesis. The fetal inflammatory response syndrome (FIRS) is described as a systemic activation of the fetal innate immune system, with or without infectious process, marked by elevation of acute phase reactants and inflammatory cytokines such as interleukin‐6 (IL‐6) in the cord blood (reviewed in Jung et al. 2020). FIRS is suggested to be an important process leading to premature birth (Jung et al. 2020) and is associated with a slew of neonatal morbidity including BPD (Mittendorf et al. 2005; Nomiyama et al. 2023). FIRS is increasingly prevalent at the lowest gestation, highlighting that a large proportion of ELGANs most at risk for BPD are born in an inflammatory environment (Nomiyama et al. 2023). Increased levels of pro‐inflammatory markers are commonly seen in conditions such as chorioamnionitis (amniotic fluid; Yoon et al. 1997, cord blood; Misra et al. 2015) likely intimately related to FIRS. Yet, exposure to FIRS may prove helpful in lung maturation and partially protective for respiratory distress syndrome (RDS) (the Watterberg hypothesis) (Jobe 1999; Watterberg et al. 1996). Administration of prophylactic physiological hydrocortisone (used for relative adrenal insufficiency) in ELGANs born from chorioamnionitis, but not the population of ELGANs as a whole, initially showed a reduction of BPD rates in survivors (Watterberg et al. 2004) in an early trial. The effect was less pronounced when extended to all ELGANs in larger studies (Baud et al. 2016; Baud and Lehert 2024). This highlights the potential dual effect of physiological hydrocortisone in supporting low endogenous cortisol and exerting an anti‐inflammatory effect in patients with in utero inflammatory exposure. It also clarifies the importance of identifying BPD endo/phenotypes to tailor therapeutic interventions.

#### 1.5.3 The Obvious, but Unmodifiable, Risk Factors of BPD

Relatively unmodifiable attributes play an important role in the risk of injury and consequent BPD. The gestational age (by proxy, the maturation and morphogenic stage of the lung) and weight at birth are the most important risk factors, with the most immature/smallest infants being most at risk for BPD (Zysman‐Colman et al. 2013). Reliance on mechanical ventilation is linearly and inversely correlated with GA (Norman et al. 2023); though the invasive ventilation days are considerably increased in infants born less than 24 weeks (median of 22 days at 24 weeks, 32 days at 23 weeks, and 54 days at 22 weeks' gestation in comparison to a median of 2 days or less for infants born 25+ weeks gestation) (Norman et al. 2023). Dependence on non‐invasive ventilation or oxygen at 36 weeks cGA is upward of 80% in infants born at 22 weeks of gestation (Norman et al. 2023). Male sex is associated with BPD and its severity (Laughon, Langer, et al. 2011; Binet et al. 2012). Genetic contributors of BPD have surfaced as potentially important factors, stemming from twin studies (reviewed in detail elsewhere; Lavoie and Rayment 2023; Leong 2019). However, strong candidates showing persistence across studies/populations have been difficult to identify (Lavoie and Rayment 2023), highlighting the important variation and polygenic factors at play. MicroRNA (miRNA) are surfacing as interesting plausible targets due to their ability to mediate numerous targets (reviewed in Leong 2019). This myriads of risk factors prime the fetal lung for subsequent development of BPD (Figure 2—*Pre‐disposing factors*).

#### 1.5.4 The Unique Circumstances of the Premature Neonatal Lung in the Acute Post‐Natal Phase of Injury and Beyond

At birth, ELGAN lungs are suddenly confronted with the necessity to carry out respiratory functions. This occurs while their lungs may only contain the most basic ventilatory units as the pulmonary acinus develops and future alveolar epithelium thins to generate the respiratory bronchioles in the late canalicular stage (Pierro, Van Mechelen, et al. 2022; Plosa and Sucre 2024). Numerous noxious stimuli are readily exposing the preterm lung to damage during the initial phase of transition to post‐natal life, where respiratory distress syndrome (or hyaline membrane disease—the acute lung injury) settles in. The premature lungs' transition from a hypoxic environment in utero to being exposed to 21+% fraction of inspired oxygen (FiO_2_). Even room air (RA) has been shown to limit lung growth in preclinical models (Stocks et al. 2013; Gebb and Jones 2003). Even when limited to the brief period of transition during resuscitation, higher FiO_2_ can lead to higher oxidative stress that can persist well over 7 days (Vento et al. 2009). Due to the ELGANs' immature gas exchange capacity, this hyperoxic stimulus is often sustained beyond the initial transition to post‐natal life (Norman et al. 2023). Oxygen therapy and resulting reactive oxygen species (ROS) are well‐established contributors to BPD pathogenesis (reviewed in Sotiropoulos and Oei 2023; Buczynski et al. 2013; Weinberger et al. 2002). Indeed, important clinical trials have focused on identifying the best oxygen saturation targets to limit over‐treatment/under‐treatment of hypoxia with oxygen therapy (STOP‐ROP Multicenter Study Group 2000; BOOST‐II Australia and United Kingdom Collaborative Groups 2016; SUPPORT Study Group of the Eunice Kennedy Shriver NICHD Neonatal Research Network 2010a). Both hypoxia and hyperoxia lead to an array of alterations in various molecular pathways (mostly affecting vascular endothelial growth factor (VEGF) and hypoxia inducible factor (HIF) signaling—reviewed in Liao and Zhang 2020; Collard et al. 2004; Schofield and Ratcliffe 2004) impacting the cooperative alveolar‐vascular development. Hyperoxia alone is the basis of important BPD preclinical models in rodents (reviewed in Berger and Bhandari 2014). These models show reduced alveolar complexity, abnormal lung vasculature, inflammation, fibrosis, and right‐sided heart strain (Berger and Bhandari 2014). Interestingly, varying intensity of oxygen exposure in murine preclinical models also leads to potentially different pathogenic responses in the developing lung. For example, mild hyperoxia conditions appear to promote airway hyperreactivity more prominently than severe hyperoxia conditions (Raffay and Martin 2020). Hyperoxia has been shown to not only affect the lungs but potentially promote a long‐term pro‐fibrogenic phenotype (1 year post‐exposure) in systemic blood vessels, heart, and kidney in preclinical models (Defreitas et al. 2024). This is in keeping with the effect on blood pressure, reduced complexity, and hyperactivity of the vasculature in adult rats exposed to hyperoxia in the neonatal period (Yzydorczyk et al. 2008). In preclinical models, hyperoxia causes lung endothelial colony‐forming cell (ECFCs) dysfunction (Baker et al. 2009; Alphonse et al. 2014). BPD patients have fewer ECFCs in cord blood compared with premature infants without BPD (Baker et al. 2012). This vascular phenotype in BPD has been extensively reviewed elsewhere (Durlak and Thébaud 2024). Beyond the vascular impact of excessive oxygen exposure, repercussions are also seen in the lung parenchyma. Major transcriptomic changes at the single cell level are observed in mesenchymal and epithelial cell populations of the developing lung in a murine hyperoxia model, with inflammation and angiogenic pathways appearing as significant drivers of disease (Hurskainen et al. 2021). Furthermore, ELGANs are ill‐equipped to face the increased production of ROS due to poor endogenous antioxidative capacity and repair mechanisms (Weinberger et al. 2002; Collard et al. 2004). This imbalance leads to peroxidation of cellular components, such as lipids and proteins, a finding commonly seen in those facing higher oxygen and ventilation needs (Gladstone Jr. and Levine 1994), ultimately altering cellular signaling and function (reviewed in Escobar et al. 2011). The pathways involved in oxidative stress and anti‐oxidative capacity in preterm patients and preclinical models have been reviewed elsewhere (Wu et al. 2024; Escobar et al. 2011).

Compounding to the instability in hypo/hyperoxic stimuli, mechanical ventilation is often required to support ineffective respiratory function in ELGANs to build functional residual capacity (FRC) (Norman et al. 2023). This is in part due to immature respiratory drive, weak musculature, and increased surface tension due to the absence/inadequacy of surfactant from immature/inexistent alveolar type 2 (AT2) cells (Buczynski et al. 2013). Although an undeniable breakthrough in neonatal care, surfactant replacement therapy has been associated with increased BPD rates (Dunn et al. 2011; SUPPORT Study Group of the Eunice Kennedy Shriver NICHD Neonatal Research Network 2010b). The drastic improvement in the management of RDS, with mortality rates declining from nearly 50% in the 1970s to less than 5% in current days (Jobe 2025; Schwartz et al. 1994), now allows the smallest ELGANs to have a chance at life (Domellof and Jonsson 2018; Edwards et al. 2024; Kono et al. 2021). Thus, while exogenous surfactant effectively overcomes the biochemical immaturity of the lung and cures RDS, it does not overcome the structural immaturity of the most immature ELGANs. Lifesaving, positive pressure ventilation is required to maintain respiratory function until further maturation can be achieved. This has been associated with BPD in numerous reports (reviewed in Keszler and Sant'Anna 2015; Lee et al. 2022) and reproduced in large animal models such as sheep and baboons (reviewed in Albertine 2015). For this reason, a large shift toward non‐invasive ventilation has occurred in ELGANs. The mechanical stretch produced by invasive mechanical ventilation (and other forms of ventilation) is invariably imperfect, leading to under‐ and over‐inflation, with frequent regional lung differences, leading to baro‐volu‐atelecto‐trauma. This further accentuates the heterogeneity of BPD, not only from patient to patient but within an individual patient's lungs as well (Gibbs et al. 2020). Mechanical “overstretch” can transduce an inflammatory cascade (Wright and Kirpalani 2011) marked by pulmonary neutrophilic infiltration, cytokine release by M1 macrophages polarization (Hurskainen et al. 2021; Cyr‐Depauw et al. 2021) and fibrosis via TGF‐β (simplified view—this topic has been reviewed extensively in Thébaud et al. 2019; Speer 2006; Shahzad et al. 2016; Wright and Kirpalani 2011). Much of these processes appears to have a predominant focal point identified as nuclear factor‐kappa B (NF‐κB) (Wright and Kirpalani 2011).

The initial events of resuscitation, bringing together multiple adverse stimuli (trauma stemming from invasive mechanical ventilation, hyperoxia, prenatal conditions) set the stage for ongoing inflammation as the acute lung injury transition into a chronic phase of injury with long‐term persistence of the adverse stimuli overlayed with post‐natal complications such as sepsis and necrotizing enterocolitis (NEC). These systemic inflammatory conditions (Collard et al. 2004; Cakir et al. 2023) (Figure 2—*Other relevant insults*) further worsen the extent of lung injury. In addition, the increased antibiotic use alters the lung microbiome, which may contribute to BPD pathogenesis (Pammi et al. 2019; Xu et al. 2022; Gao et al. 2024; Shi et al. 2024). The consequences of this array of harmful stimuli at the lung micro‐anatomic level are often difficult to appreciate clinically. Rather, the clinician is mostly restricted to appreciate the evolving BPD pathogenic process through the lens of oxygenation and ventilation parameters. This leaves neonatologists poorly equipped to manage these patients as these outcomes can also vary by situations or maturation stages. While the use of molecular markers remains uncommon (Wright and Kirpalani 2011) in the clinic, there is an increasing body of evidence highlighting potentially useful markers for adoption (reviewed in Wright and Kirpalani 2011; Cui and Fu 2023; Xu et al. 2023). These markers may help to differentiate BPD phenotypes and endotypes, allowing for personalized approaches and management (Pierro, Van Mechelen, et al. 2022).

## 2 Mesenchymal Stromal Cells as a Potential Therapy for the Heterogeneous Neonatal Lung Injury Leading to the Development of BPD

Identifying a versatile therapeutic agent for the heterogeneous BPD phenotype is arduous due to the wide variability of susceptible patients and incurring insults. The pathogenic course of each patient will be unique. The ideal therapeutic candidate will be an agent that can offer a wide therapeutic scope. Ideally, it would adapt to the lung microenvironment in real time. Mesenchymal stromal cells could fit this therapeutic profile.

### 2.1 Mesenchymal Stromal Cells: Another Definition Controversy

MSCs were initially identified as bone marrow‐derived colony‐forming unit‐fibroblast with critical supporting functions to the hematopoietic stem cells microenvironment (Friedenstein et al. 1976). Their historical ascension as the “favorite cell type” of the cell therapy research community has been reviewed on numerous occasions (Thébaud 2018, 2023; Galipeau and Sensebe 2018; Pittenger et al. 2019). Renamed MSCs by Caplan et al. (1991), these cells were found to have the ability to differentiate into mesoderm‐derived cells such adipose, bone, and cartilaginous cells in vitro. MSCs have since been found in many compartments/organs of the human body, generating a wealth of potential MSCs' sources, with potential varied intrinsic capacities (Caplan 2017). Extensive reviews of their origins and functions have been published elsewhere (Pittenger et al. 2019; Caplan 1991, 2010; Caplan and Dennis 2006). The excitement generated toward these cells as a potential cell therapy came at the expense of poor cell characterization reporting in published research (Renesme, Pierro, et al. 2022), putting them at risk of misuse, mislabeling, and inadequate reporting, blurring their true therapeutic potential (Wiest 2025). Thus, the International Society of Cell & gene Therapy (ISCT) defined the minimal criteria for MSCs which, under culture conditions, had (1) the ability to adhere to plastic surface, (2) differentiate in osteoblast, adipocytes and chondroblasts, (3) display the expression of cell surface markers CD105, CD73 and CD90 while lacking CD45, CD34, CD14/CD11b, CD79α/CD19, and HLA‐DR (Dominici et al. 2006). However, significant debates about their definition and naming convention continue among the MSCs' research community (as highlighted in recent opinion pieces; Caplan 2017; Wiest 2025; Renesme, Cobey, et al. 2022). Indeed, appropriate reporting of the cell product in use (or in other words, “the therapy”) remains poor, only occurring in 1 in 5 studies, de‐stabilizing the foundation researchers are attempting to build to translate them (Renesme, Pierro, et al. 2022; Maltais‐Bilodeau et al. 2025). A Delphi approach to establish a consensus definition for MSCs and reporting guidelines for MSC‐based clinical trials may lead to improved transparency and reproducibility in the conduct and reporting of MSC research (Renesme et al. 2025).

### 2.2 MSC as a Cell Therapy: Where Is All the Hype Coming From?

Over the years, MSCs became an appealing cell population that could allow cellular therapy to become a reality (Pittenger et al. 2019; Caplan 1991; Caplan and Dennis 2006). Yet, clinical trials did not measure up to the promising results in preclinical studies (Galipeau and Sensebe 2018). Condensing 50 years of research, MSCs' characteristics are conducive to the feasibility of in‐human trials (reviewed in Galipeau and Sensebe 2018; Pittenger et al. 2019). They are easy to culture, facilitating scalability and production while harboring an acceptable safety profile, immunogenic potential, and lack of engraftment without clear long‐term oncogenic capacity (despite initial concerns of the research community) (Galipeau and Sensebe 2018). Indeed, the initial therapeutic promises expected to be cell replacement at the site of injury were unlikely as MSCs engraftment is minimal (Pittenger et al. 2019; Hansmann et al. 2012). Rather, a transient bystander effect where MSCs sensed the injury environment to provide beneficial signals and factors through paracrine and cell‐to‐cell contact was backed by numerous emerging reports (Caplan and Dennis 2006; Xu et al. 2007; Van Haaften et al. 2009; Aslam et al. 2009; Ortiz et al. 2003). MSCs had clear anti‐inflammatory effects (Xu et al. 2007; Ortiz et al. 2007). Many reports also highlight their regenerative properties marked by alveolar repair (Curley et al. 2012; Pierro et al. 2013), pro‐angiogenic (Sharma et al. 2022; Zhou et al. 2022), and anti‐fibrotic (Moodley et al. 2009) abilities among others (reviewed; Pittenger et al. 2019; Caplan and Dennis 2006; Stavely and Nurgali 2020; Giacomini et al. 2023; Han et al. 2022). Adding to their paracrine factor release, it was recently proposed that their demise (occurring within the first hours following their administration; Pang et al. 2021) is thought to drive some of the beneficial effect observed in preclinical models (Pang et al. 2021). The concept that their released cargo (such as extracellular vesicles [EVs]), rather than the MSCs themselves, is exerting the therapeutic effect raised the possibility that their secretome/EVs could be a new “cell‐free” therapeutic strategy (MSCs' secretome has been reviewed elsewhere; Han et al. 2022; Kou et al. 2022; Lesage and Thébaud 2022). Despite more than 55,000 published studies as of a report in 2019 (Pittenger et al. 2019) and over 1000 clinical trials registered on clinicaltrials.gov (Pittenger et al. 2019; Han et al. 2022), MSCs have reached a dismal success rate with clinical translation and licensing. Market authorization has been obtained for only two indications: Graft Versus Host Disease (GHVD) and Crohn's‐related enterocutaneous fistular disease in a handful of countries (Galipeau and Sensebe 2018; Pittenger et al. 2019). Endeavors to test MSCs in clinical trials should be backed with a strong rational and biological plausibility.

### 2.3 Establishing Biological Plausibility of MSCs as a Treatment for BPD

MSCs, particularly umbilical cord (UC) derived, revealed to be an appealing option for ELGANs at risk for BPD. First, they are available from the otherwise discarded umbilical cord of the mother. Second, MSCs can have the capacity to provide a tailored therapeutic response to BPD's heterogeneous pathogenic process and appear to specifically travel to the site of injury (Xu et al. 2007; Van Haaften et al. 2009; Ortiz et al. 2003; Moodley et al. 2009). Third, their regenerative/repair capacity could optimize functional and architectural lung development, closing the gap with normally developed lungs.

### 2.4 MSCs and Their EVs Promote Lung Development and Limit Injury in Preclinical Models of BPD

Numerous studies suggest lung protective effects of MSCs and their EVs in experimental neonatal models mimicking BPD. The culmination of these studies has been systematically reviewed on several occasions in recent years (Augustine et al. 2017, 2020). Despite significant heterogeneity between preclinical studies, MSCs improve various endpoints, including alveolarization, lung vascular growth, inflammation, pulmonary hypertension, apoptosis, and fibrosis in meta‐analysis (Augustine et al. 2017). MSCs also improve long‐term pulmonary function (Willis et al. 2018) and exercise capacity (Hansmann et al. 2012). Considering other cell types for BPD treatment, MSCs are the leading cell therapy showing superior strength of their effect (Augustine et al. 2020). However, the meta‐analysis underscored the heavy reliance on rodent models and lack of large animal model use in MSC research for the treatment of BPD (Augustine et al. 2017). Since, few reports of the use of MSCs in large animal models recreating the preterm lung environment have been published. In chronically ventilated preterm baboons, MSCs failed to show benefits on lung structure and function but showed improved cardiorespiratory stability in those treated with MSCs (Mobius et al. 2023). In contrast, isolated EVs from human bone marrow‐derived MSCs led to improved physiological parameters (respiratory severity score, saturation/FiO_2_ [S/F] ratio, A‐a gradient), alveolar network complexity, and reduced smooth muscle accumulation in preterm sheep invasively ventilated for 7 days (Albertine et al. 2024).

**FIGURE 3:**
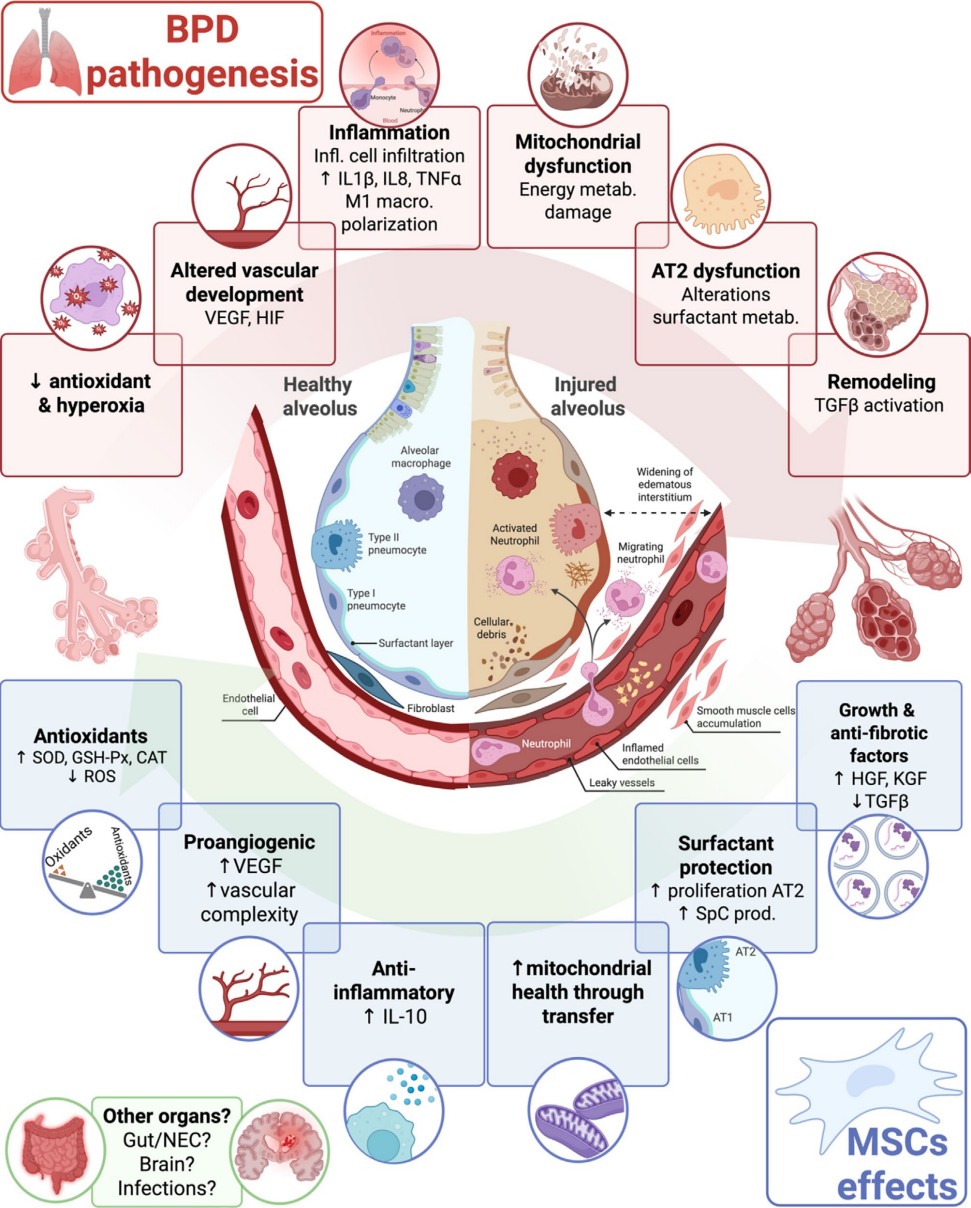
MSC mechanisms in the context of BPD pathogenic processes. A depiction of major pathways involved in BPD pathogenesis is featured at the top of the figure while MSCs counter actions are featured at the bottom of the figure. More details concerning each box are present in the body of the manuscript. ↓, reduce; ↑, increase; BPD, bronchopulmonary dysplasia; macro., macrophage; metab, metabolism; NEC, necrotizing enterocolitis; prod: production. Created in BioRender. Deguise M. (2025) https://BioRender.com/kqik256 (modified from BioRender (2025) Acute Respiratory Distress Syndrome template).

The molecular mechanisms driving the beneficial effects of MSCs and their secretome (MSCs/EVs is used to simplify the text and represent and/or) on BPD pathogenic pathways (reviewed in detail in Thébaud 2018, 2023; Lesage and Thébaud 2022; Tang et al. 2022; Zhang et al. 2023; Ee and Thébaud 2018; Lesage and Thébaud 2018; Liu et al. 2022) provide the rationale as a therapeutic intervention for BPD. A simplified schematic of the various functions of MSCs in the context of BPD pathogenesis is depicted in Figure 3.

#### 2.4.1 Anti‐Inflammatory Properties

MSCs/EVs have pleiotropic effects on both the innate and adaptive immune system, blunting both early and late pathogenic events in BPD. MSCs/EVs reduce neutrophilic infiltration likely due to their modulation of the cytokine release (Ortiz et al. 2007; Curley et al. 2012; Moodley et al. 2009). They can partially restore lung levels of IL‐1β, IL‐6, IL‐10, TNFα, macrophage inflammatory protein‐1α (MIP‐1α), monocyte chemoattractant protein‐1 (MCP‐1), among others (Sharma et al. 2022; Zhou et al. 2022; Chou et al. 2016), via a paracrine mechanism. These cytokines have been associated with BPD pathogenesis. MSCs are also attracted to injured lung cells in vitro, and cell‐to‐cell contact amplifies the normalization of cytokines such as MIP‐1α and RANTES (Xu et al. 2007). On the other hand, MSCs also promote anti‐inflammatory signals such as IL‐10 (Curley et al. 2012), which potentially alters the cellular migration of pro‐inflammatory cells. Numerous reports suggest macrophage‐dependent immunomodulation by MSCs/EVs, leading to a shift away from the M1 inflammatory macrophages to preferential polarization akin to the M2 anti‐inflammatory profile (Willis et al. 2018; Morrison et al. 2017; Wang et al. 2020). This appears to be mediated by the uptake of exosomes (Willis et al. 2018). Some have proposed that this transition occurs as M1 macrophages phagocytose MSCs (Harrell et al. 2019). When it comes to the adaptive immune system, MSCs/EVs also effectively modulate T‐cell physiology. Their secretion of IL‐1RN, an inhibitor of IL‐1, can limit T‐cell response to IL‐1 signals in vitro, and suppress the inflammatory cytokines IL‐1 and TNF‐α in a model of acute lung injury (Ortiz et al. 2007). MSCs drive an anti‐inflammatory environment by restoring the balance of inflammatory Th17 T‐cells and anti‐inflammatory regulatory T‐cells (Tregs) by favoring a shift toward Tregs fate (Wang et al. 2019). It is thought that MSCs also have a polarization capacity in response to their environment, in which they assume an anti‐inflammatory phenotype when facing a large inflammatory stimulus (reviewed in Liu et al. 2022). This may underlie the basis of their potent anti‐inflammatory actions but also explain the variable response observed in some studies due to the heterogeneity of the model systems. As such, MSCs and their secretome appear to provide important regulation all along the evolving inflammatory injury seen in BPD.

#### 2.4.2 Antioxidative Capacities

Given the poor anti‐oxidative response of ELGANs and the knowledge that oxygen therapy can significantly drive free radicals' formation, uplifting compensatory mechanisms is essential to limit damage from oxidative stress. MSCs roles in oxidative stress have been recently reviewed elsewhere (Stavely and Nurgali 2020). Specific to lung injury models, MSCs can promote the activity of antioxidants such as superoxide dismutase (SOD), glutathione peroxidase (GSH‐Px), and catalase (CAT) in a rodent LPS‐induced lung injury model (Lei et al. 2021). Consequently, upon MSC treatment, there is reduced ROS production and reduced content of protein carbonyl, a finding related to damaged protein exposed to ROS (Lei et al. 2021). This mechanism appears directly related to Nuclear factor‐erythroid 2 p45‐related factor 2 (NRF2) and hemeoxygenase 1 (HO‐1) pathway, as their blockade reduced the effect of MSCs in vivo (Lei et al. 2021). Comparable findings were obtained when using MSC‐conditioned media (CM) in a similar rodent model of ALI, where the attenuation of oxidative stress was attributed to NRF2/NF‐κB/HO‐1 signaling (Tang et al. 2021). In vitro, MSC‐CM can limit apoptosis of hypoxic alveolar epithelial cells by limiting ROS production, enhancing antioxidation, and limiting expression of cell death effector protein usually regulated by ROS and hypoxia inducible factor (HIF)‐1 (Bernard et al. 2018). Interestingly, priming MSCs with hyperoxia before injection of their MSC‐CM in diseased animals leads to improvements in lung structure and pulmonary hypertension (Waszak et al. 2012). O_2_‐primed MSCs‐CM had enhanced expression of antioxidant stanniocalcin‐1 (STC‐1) (Waszak et al. 2012). Priming MSCs with other antioxidants such as HO‐1 leads to superior therapeutic effects in a rodent LPS‐induced ALI model (Chen et al. 2019). Such enhancement of MSCs could be harnessed as a bioengineering tool to enhance MSCs/EVs therapeutic effect in future clinical trials.

#### 2.4.3 Pro‐Angiogenic Properties

MSCs/EV treatment promotes lung vascular growth and attenuates pulmonary hypertension (Van Haaften et al. 2009; Sharma et al. 2022; Willis et al. 2018). MSCs enhanced VEGF and the pro‐angiogenic transcriptome, leading to an improved pulmonary vascular network in histological studies (Sharma et al. 2022; Zhou et al. 2022; Willis et al. 2018; Chou et al. 2016). Ablation of VEGF by gene knockdown in MSCs before their delivery leads to reduced therapeutic capacity in rodent hyperoxia or LPS‐induced models (Yang et al. 2016; Ahn, Park, et al. 2018). Following MSCs/EVs administration, an improved capillary network is often associated with attenuated right heart strain (Van Haaften et al. 2009; Sharma et al. 2022; Zhou et al. 2022).

#### 2.4.4 Mitochondrial Transfer

In 2006, a report provided evidence that MSC could transfer mitochondria in co‐culture conditions with an immortalized lung cell line that had been depleted of their mitochondrial component (Spees et al. 2006). This allowed for the restoration of aerobic cellular function and reduction of ROS in the recipient cells (Spees et al. 2006). This led to the important premise that MSCs may be able to support mitochondrial function of resident tissue by active transfer of mitochondria to cells that may have been overburdened by hyperoxia and ROS. This concept has been at the center of active investigations as mitochondria are central to numerous biological processes, including mitochondrial mass homeostasis (fission, fusion, mitophagy), energy production, ROS production, cell death, and immune modulation via mitochondrial transfer to immune cells (reviewed in Han et al. 2020). Hyperoxia can significantly hinder these processes (reviewed in Xuefei et al. 2021). Mitochondrial transfer has been observed in numerous cell types relevant to BPD pathogenesis, such as macrophages, alveolar epithelial cells, and vascular endothelial cells (Morrison et al. 2017). Relevant to MSC biology in BPD, this appears to occur via tunneling nanotubes, cell‐to‐cell interactions, and EVs (Han et al. 2020). In an LPS‐induced acute respiratory distress mouse model, administration of MSCs harboring dysfunctional mitochondria did not show a beneficial anti‐inflammatory effect (Morrison et al. 2017). Similarly, MSC recipient mice without a nascent functioning oxidative phosphorylation do not show benefits from MSC treatment (Morrison et al. 2017). Mitochondrial transfer appears essential to the induction of the anti‐inflammatory M2 macrophage, a process relying on oxidative bioenergetics (Morrison et al. 2017). This highlights the role of mitochondrial oxidative respiration as an important player mediating MSCs' effect (Morrison et al. 2017).

#### 2.4.5 Anti‐Fibrotic Abilities

MSCs dampen pathways driving fibrogenesis in lung injury models, such as transforming growth factor‐β (TGF‐β) at both the mRNA and protein levels, potentially via Smad2 transcriptional factor (Moodley et al. 2009). MSCs also modulate the turnover of collagen deposition by fostering a pro‐degradation profile between matrix metalloproteinases (MMP) and their endogenous inhibitors (Moodley et al. 2009). To note, MMPs are commonly downregulated post‐MSC administration (Ortiz et al. 2007; Moodley et al. 2009). As such, it is possible that MSCs may act through other pathways. For example, MLE‐12 cells (a type of AT2 cell line) exposed to LPS display enhanced NF‐κB/Hedgehog‐mediated expression of fibrotic markers, which can be reversed by MSCs/EVs through microRNA cargo (Xiao et al. 2020). These findings could be reciprocated in an in vivo rodent ALI model (Xiao et al. 2020).

#### 2.4.6 Beneficial Growth Factors Release

MSCs release growth factors that can amplify their regenerative capacities (Tang et al. 2021). For example, alveolar epithelial cells exposed to MSC‐CM enhanced wound closure by scratch assay (Van Haaften et al. 2009; Curley et al. 2012). When the media is treated with keratinocyte growth factor (KGF) antibody, it partially blunts the beneficial effect observed (Curley et al. 2012). Cell death and HIF‐1 expression in hypoxic alveolar epithelial cell culture models are reduced by the presumed release of KGF and hepatocyte growth factor (HGF) present in MSC‐CM (Bernard et al. 2018). Co‐cultures of LPS‐exposed pulmonary vascular endothelial cells treated with MSCs‐EVs had improved permeability, viability, and inflammatory profile dependent on HGF expression (Wang et al. 2017). Of interest, priming of MSCs by overexpression of HO‐1 harbors enhanced capacity for growth factor production of KGF and HGF in rodent ALI models (Chen et al. 2019). It is likely that the abundance of growth factors released by MSCs has further regenerative capacity that has yet to be uncovered.

#### 2.4.7 AT2 Cells and Surfactant Protection

AT2 cells play a central role in RDS and ALI given their role in surfactant production. Their transcriptome and surfactant metabolism appear impacted in the hyperoxia (Hurskainen et al. 2021). They appear as an important target of MSCs. MSCs/EVs allow for enhanced AT2 proliferation capacity, maintenance of their population number as well as surfactant protein SpC production (Wang et al. 2019), stabilizing pulmonary lung dynamics, likely stemming from a presumed improved alveolar patency in models of lung injury (Zhou et al. 2022).

### 2.5 Targeting Organ Crosstalk as a Side Effect of MSCs Treatment for BPD

MSCs' pleiotropic effects indicate potential benefits on other organ systems. Indeed, MSCs are being considered for a whole array of neonatal conditions beyond the respiratory system, including intraventricular hemorrhage (Ahn et al. 2013; Ahn, Chang, et al. 2018), hypoxic–ischemic encephalopathy (Mintoft et al. 2024), necrotizing enterocolitis (Maltais‐Bilodeau et al. 2025), among others (reviewed in Paton et al. 2025; Malhotra et al. 2023; Ahn, Park, et al. 2021). Recent reports have also been able to link BPD pathogenic processes and neurodevelopmental impairments in preclinical models (Lithopoulos, Toussay, et al. 2022). Interestingly, MSCs improved the number of Sox2/Nestin1 neural progenitor cells and enhanced their capacity for self‐renewal (Lithopoulos, Strueby, et al. 2022). In addition, MSCs have shown positive effects in preclinical models of sepsis, a common complication seen in premature infants (Zhu et al. 2017). Such findings strengthen the possibility that MSCs treatment in preterm infants at risk of BPD is likely to have far‐reaching effects beyond the lungs.

## 3 Translating MSCs From the Bench to the Bedside: Where Are We Now?

### 3.1 Clinical Trials of MSCs for the Treatment of BPD

**FIGURE 4:**
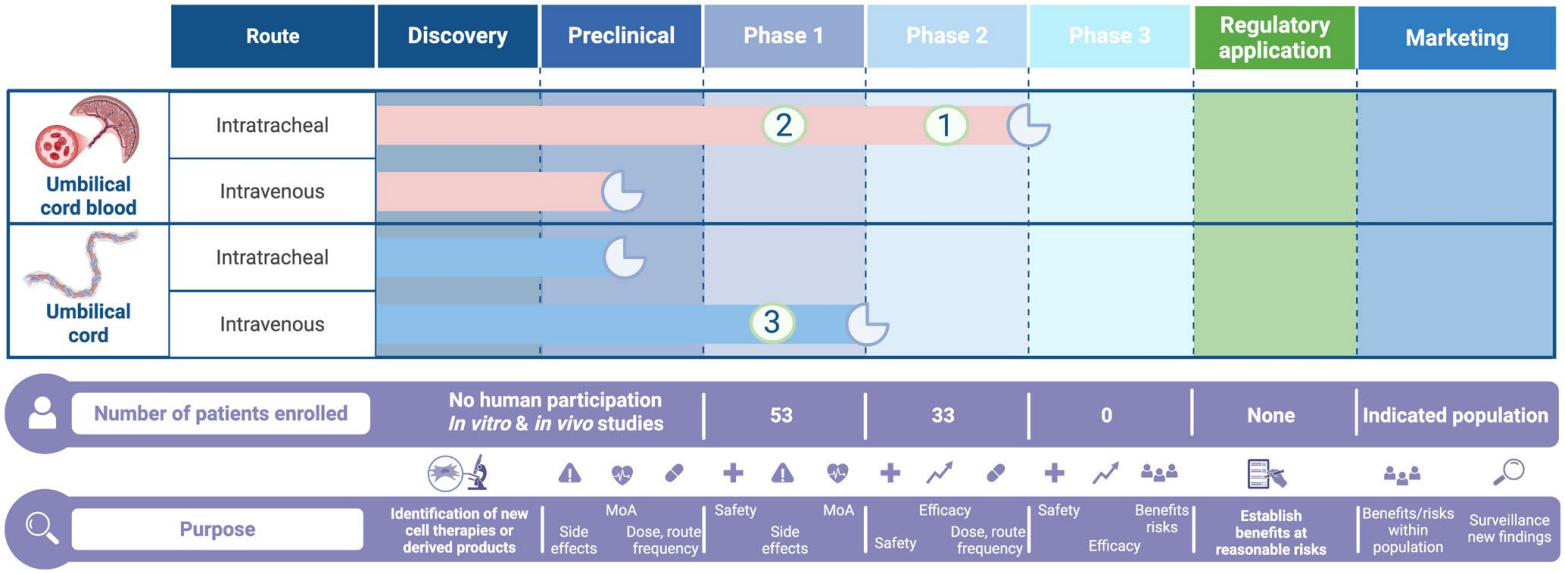
Current MSC cell therapy development pipeline with an overview of completed clinical trials for BPD. The number in the circle represents the number of trials that have been completed. The incomplete circle represents the current stage of development. The total number of patients in each phase is depicted below the table of clinical trials progress. The general purpose of each phase is described in the bottom strip with complementary pictograms. Note that MSCs from other sources may be in pre‐clinical development but have not been used in clinical trials with published results. They are not represented in this figure for the sake of clarity and simplification. MoA: mechanism of action. Created in BioRender. Deguise, M. (2025) https://BioRender.com/4pe8df6.

In 2017, a first Cochrane review identified a few phase I clinical trials using MSCs in preterm infants at risk of developing BPD (Pierro et al. 2017). Since then, more phase I/II clinical trials have emerged. We describe these studies in detail in Table 1. An overview of current translational efforts is provided in Figure 4. Briefly, MSCs appear as a safe biological product with no serious adverse events that have been directly attributed to MSCs. The longest follow‐up to date for safety is up to two years (Ahn et al. 2017). The authors reported safety and no tumorigenicity by the end of their study (Ahn et al. 2017). Clinical trials to date have mainly focused on MSCs derived from the umbilical cord, with blood and tissue being the most common sources. Interestingly, umbilical cord blood‐derived MSCs (UCb‐MSCs) have been administered only via the intratracheal (IT) route (Ahn et al. 2017, 2023; Chang et al. 2014; Ahn, Chang, et al. 2021; Powell and Silvestri 2019) while umbilical cord‐derived MSCs (UC‐MSCs) have preferentially been administered intravenously (IV) (Xia et al. 2023; Thebaud et al. 2024; Marín et al. 2024; Nguyen et al. 2020; Álvarez‐Fuente et al. 2018) (Table 1). Doses have ranged widely from 1 × 10^6^ to 2 × 10^7^ cells/kg (Table 1) and most studies only had one administration timepoint (Ahn et al. 2017, 2023; Chang et al. 2014; Ahn, Chang, et al. 2021; Powell and Silvestri 2019; Xia et al. 2023; Thebaud et al. 2024). Safety in repeated MSC administrations protocol was shown in three studies (Marín et al. 2024; Nguyen et al. 2020; Álvarez‐Fuente et al. 2018). Interestingly, nearly all trials administered MSCs within the first three weeks of life (Ahn et al. 2017, 2023; Chang et al. 2014; Ahn, Chang, et al. 2021; Powell and Silvestri 2019; Thebaud et al. 2024; Marín et al. 2024).

**TABLE 1:** Update of currently published reports/clinical trials of mesenchymal stromal cells for BPD.

Study	Population	Cells	Admin	Outcomes	Findings
Type/phase; # of center; (clinicaltrials.gov)	GA; *N*; Features	Dose; ISCT crit.; Function	Route; Time	1°; 2°	
**Umbilical cord blood‐derived MSCs**					
Chang et al. (2014) and Ahn et al. (2017) Phase I dose escalation One center (NCT01297205) (NCT01632475)	23–29 weeks *N* = 9 At risk BPD/on vent. PND 5–14	UCb‐MSCs 1 × 10^7^/kg 2 × 10^7^/kg No ISCT No Fxn	IT PND5‐14	1°: Safety 2°: Feasibility, efficacy (BPD, inf. markers), long‐term safety	1°: Well‐tolerated, no SAE recorded related to MSCs 2°: ↓ Severity of BPD?, no home O_2_ inf. cytokine reduced No tumorigenicity at 2 years of age higher body weight at 18–24 months versus historical ctrl No NDI in MSCs group versus historical ctrl
Ahn, Chang, et al. (2021) and Ahn et al. (2023) Phase II double blind Block randomized Two centers (NCT01828957) (NCT01897987)	23–28 weeks *N* = 33 At risk BPD/on vent. PND 5–14	UCb‐MSCs 1 × 10^7^/kg No ISCT No Fxn	IT PND5‐14	1°: Death or mod/severe BPD *5 years FU* Composite resp. morbidity over 5 years 2°: inf. cytokines prem. complications *5 years FU* Individual resp. morbidities, rate of CP and NDI	1°: No Δ in death or mod/severe BPD *5 years FU* Trend toward less composite resp. morbidities compared with control group, not statistically significant, mostly driven by GA25–28 patients 2°: ↓ severity BPD in 23–24 weeks GA (underpowered)? inf. cytokine reduced No adverse events until 6 months *5 years FU* Less ER visit in MSCs group, O_2_ therapy and possibly developmental delay, mostly driven by GA25–28 patients
Powell and Silvestri (2019) Phase I dose escalation One center (NCT02381366)	< 28 weeks, < 1000 g *N* = 12 At risk BPD/on vent. PND 5–14	UCb‐MSCs 1 × 10^7^/kg 2 × 10^7^/kg **Partial ISCT** No Fxn	IT PND6‐14	1°: AE within 84 days 2°: AE 84 days to 20 months Rate mod/severe BPD or death at 36 weeks PMA Hospital admission 84 days to 20 months Bayley 84 days to 20 months	1°: Well‐tolerated, no SAE recorded related to MSCs 2°: high BPD rates 12/12 patients, severe BPD 10/12 patients, no data on Bayley or admission
**Umbilical cord tissue‐derived MSCs**					
Xia et al. (2023) Phase I dose escalation Open label One center (NCT03558334)	No max GA *N* = 13 Severe BPD (28 days 0.3 FiO_2_ or PPV)	UC‐MSCs 1 × 10^6^/kg 5 × 10^6^/kg No ISCT No Fxn Freshly produced cells	IV Variable < 32 weeks: 36 weeks cGA or DC > 32 weeks: PND56 or DC	1°: Safety within 24 h 2°: Change in chest CT within 2 years Change in temp, BP, HR, RR, SaO_2_, growth velocity	No pre‐specified infusion‐related event (most infants 30 weeks GA—infusion time not specified) 2°: Better Silverman Andersen Score compared with pre‐infusion (no control group)
Marín et al. (2024) Phase I Dose escalation Open label Multicenter—4	< 28 weeks, < 1250 g *N* = 10 At risk BPD/on vent. With FiO_2_ > 0.3 PND 7–14	WUC‐MSCs 5 × 10^6^/kg weekly X3 No ISCT No Fxn Thawed	IV (repeated) PND16.6	1°: Safety of repeated injection 2°: Feasibility, inf. cytokines, prem. complications	1°: Well‐tolerated, no SAE recorded 2°: Similar outcome to comparison group with high BPD rates (100%) No death in MSCs treated compared with comparison group
Thebaud et al. (2024) (completed in 2023—not published—limited to abstract) Phase I open‐label, multicenter dose escalation (NCT04255147)	< 28 weeks, *N* = 9 At risk BPD/on vent. With FiO_2_ > 0.3 PND 7–28	UC‐MSCs 1 × 10^6^/kg (3) 3 × 10^6^/kg (3) 5 × 10^6^/kg (3)	IV 19.3 ± 6.1 days	1°: Safety 2°: Feasibility, efficacy (BPD, inf. markers), long‐term safety	1°: No safety concerns noted. 2°: All patients had BPD, but high‐dose patient did not have severe BPD compared with low‐dose group 2‐year follow‐up pending
**Case studies**					
Nguyen et al. (2020) Case study/phase I One center	*N* = 4 Established severe refractory BPD dependent on O_2_ therapy less than 1 year of age	UC‐MSCs 1 × 10^6^/kg weekly X2 **ISCT described** No Fxn	IV (repeated) Varied (144–173 days)	1°: Safety, respiratory improvement	1°: MSCs infusion well‐tolerated over 12 months, with observed “recovery” from BPD 2°: Reduction of fibrosis by CT scan *Authors' note*: **Good reporting of MSCs handling, characteristics, testing. Functional analysis lacking
Álvarez‐Fuente et al. (2018) Case study—off‐label One center Dose escalation	*N* = 2 Established severe BPD	BM‐MSCs 1.1 × 10^6^/kg to 1.39 × 10^7^/kg No ISCT No Fxn	IV (repeated) Varied (85 days & 5mo)	1°: Safety, feasibility, respiratory improvement 2°: inf. cytokines	1°: MSCs infusion well‐tolerated Positive changes in respiratory measures upon MSC tx, with worsening when tx stopped 2°: Change in blood mRNA inf. markers but only two patients

Abbreviations: 1°, primary; 2°, secondary; AE, adverse event; BM, bone‐marrow; BP, blood pressure; BPD, bronchopulmonary dysplasia; Crit., criteria; CT, computed tomography; DC, discharge; ER, emergency room; FU, follow‐up; Fxn, function; g, gram; GA, gestational age; HR, heart rate; inf., inflammatory; ISCT, International Society of Cell & gene Therapy; IT, intratracheal; IV, intravenous; kg, kilogram; MSC, mesenchymal stromal cell; N, number; NCT, national clinical trial number; NDI, neurodevelopmental impairment; O_2_, oxygen; PMA, postmenstrual age; PND, post‐natal day; prem., prematurity; RR, respiratory rate; SAE, serious adverse event; SaO_2_, arterial oxygen saturation; Tx, treatment; UCb, umbilical‐cord blood; Vent, ventilator; WUC, wharton jelly umbilical cord; Δ, change.

Efficacy evaluation has been either limited or underpowered. Only one phase II trial is currently available to date (Ahn, Chang, et al. 2021). In this cohort of 66 infants, the authors claimed a potential reduction in the severity of BPD in patients of the lowest gestational age group (23–24 weeks of gestation—though still underpowered for this subgroup) unlike infants of 25–28 weeks (Ahn, Chang, et al. 2021). Evaluation of cytokines in tracheal aspirate showed the possible anti‐inflammatory effect of MSCs in some studies (Malhotra et al. 2023; Chang et al. 2014) but the conclusions remain unclear. In fact, these results could be clouded by steroids administration commonly used in this population to facilitate extubation, which could affect interpretation. No efficacy studies to date have provided a head‐to‐head comparison on the mode of administration, cell source, and dosing regimen in relation to the therapeutic effect of MSCs. Importantly, future trials should adhere to recently suggested clinical reporting guidelines to enhance the transparency and quality of cell therapy trials (Renesme et al. 2025). Few studies provided adequate information about the cell product under study (Table 1).

Overall, the early translation of MSCs as a cell therapy for BPD patients, with promising results for safety in this patient population, provides an initial foundation for researchers, clinicians, and trialists to bolster their efforts toward efficacy studies. However, precipitating MSC trials may lead to translation failure. A large void remains to perfect the conditions for the best therapeutic efficacy of MSCs, including logistics of manufacturing, pre‐injection conditioning, functional testing, quality control, logistics of administration (donor, tissue, cells, dose, route), careful patient selection, and outcomes selection.

## 4 Conclusion

BPD is a devastating condition with important long‐lasting consequences. Despite improving neonatal care, its rate remains stagnant. The ever‐evolving and heterogeneous pathogenesis has made it a challenging entity to treat. MSCs' molecular versatility as real‐time sensing cells and personalized cargo delivery is biologically appealing and an exciting new developing therapy. The translation of MSCs in BPD remains in its infancy. Much of the work lies ahead to further optimize, quality control, and enhance the potential benefits of MSCs to curb BPD progression while pursuing careful, intentional, thoughtful clinical trial design to assess its effects in infants that will benefit the most.

## Author Contributions

**Marc‐Olivier Deguise:** writing – original draft and writing – review and editing. **Bernard Thébaud:** writing – original draft and writing – review and editing.

## Ethics Statement

The authors have nothing to report.

## Conflicts of Interest

The authors declare no conflicts of interest.

## Data Availability

Data sharing not applicable to this article as no datasets were generated or analysed during the current study.
